# Probiotics and Prebiotics: Any Role in Menopause-Related Diseases?

**DOI:** 10.1007/s13668-023-00462-3

**Published:** 2023-02-07

**Authors:** Luigi Barrea, Ludovica Verde, Renata Simona Auriemma, Claudia Vetrani, Mauro Cataldi, Evelyn Frias-Toral, Gabriella Pugliese, Elisabetta Camajani, Silvia Savastano, Annamaria Colao, Giovanna Muscogiuri

**Affiliations:** 1 Dipartimento di Scienze Umanistiche, Centro Direzionale, Università Telematica Pegaso, Via Porzio, isola F2, 80143 Naples, Italy; 2 Centro Italiano per la cura e il Benessere del Paziente con Obesità (C.I.B.O), Department of Clinical Medicine and Surgery, Endocrinology Unit, University Medical School of Naples, Via Sergio Pansini 5, 80131 Naples, Italy; 3 Dipartimento di Medicina Clinica e Chirurgia, Unità di Endocrinologia, Diabetologia e Andrologia, Università degli Studi di Napoli Federico II, Via Sergio Pansini 5, Naples, 80131 Italy; 4 Section of Pharmacology, Department of Neuroscience, School of Medicine, University of Naples Federico II, Naples, Italy; 5 Universidad Católica Santiago de Guayaquil, Av. Pdte. Carlos Julio Arosemena Tola, Guayaquil, 090615 Ecuador; 6 Department of Human Sciences and Promotion of the Quality of Life, San Raffaele Roma Open University, 00166 Rome, Italy; 7 Cattedra Unesco “Educazione alla salute e allo sviluppo sostenibile”, University Federico II, Naples, Italy; 8 Department of Public Health, University of Naples Federico II, Naples, Italy

**Keywords:** Menopause, Dysbiosis, Menopause-related diseases, Probiotic therapy

## Abstract

**Purpose of Review:**

The aim of this review is to provide an overview of the menopause-related changes in microbiota and their role in the pathogenesis of menopause-related diseases. In addition, evidence on probiotic supplementation as a therapeutic strategy is discussed.

**Recent Findings:**

The human microbiota is a complex community that lives in a mutualism relationship with the host. Menopause is associated with dysbiosis, and these changes in the composition of microbiota in different sites (gut, vaginal, and oral microbiota) might play a role in the pathogenesis of menopause-related diseases (i.e., osteoporosis, breast cancer, endometrial hyperplasia, periodontitis, and cardiometabolic diseases).

**Summary:**

The present review highlights the pivotal role of microbiota in postmenopausal women health, in particular it (a) may increase intestinal calcium absorption thus preventing osteoporosis, (b) is associated with reduced risk of breast cancer and type 1 endometrial hyperplasia, (c) reduces gingival inflammation and menopausal periodontitis, and (d) beneficially affects multiple cardiometabolic risk factors (i.e., obesity, inflammation, and blood glucose and lipid metabolism). However, whether oral probiotic supplementation might be used for the treatment of menopause-related dysbiosis requires further clarification.

## Introduction

Microbiota consists of a community of microbes (bacteria, fungi, and viruses) that live inside and outside of the human body [1]. In the gut, microbial species live in a harmonic symbiosis with the host, contributing to [2] (1) increase the metabolic ability to ferment indigestible carbohydrates; (2) produce vitamins, i.e., B2, B12, K, and folic acid; (3) protect against the colonization of pathogenic bacteria; and (4) promote the maturation of immune cells and the normal development of their functions, as well as the inhibition of toxins and carcinogens [3]. According to microbial taxonomy at the *phylum* level, the following gut bacteria have been identified: *Firmicutes* (60–80%, i.e., *Ruminococcus*, *Clostridium*, *Lactobacillus*, *Enterococcus*), Bacteroidetes (20–30%, i.e., *Bacteroides*, *Prevotella*, *Xylanibacter*), *Actinobacteria* (less than 10%, i.e., *Bifidobacterium*), and *Proteobacteria* (less than 1%, i.e., *Escherichia*, *Enterobacteriaceae*) [3, 4]. Nevertheless, the composition of gut microbiota may change according to host-related factors (age, gender, latitude, ethnicity, diseases) [5], lifestyle (physical exercise, habitual diet, use of prebiotics and/or probiotics), and antibiotic therapy [4, 6, 7]. Dramatic changes of the composition of gut microbiota—known as dysbiosis—have been appointed as major contributors to several diseases such as asthma [8], eczema [8], obesity [9], type 2 diabetes [10], non-alcoholic fatty liver disease [11], colon cancer [12], heart disease [13], and neurological or neuropsychiatric diseases [14]. Among factors that can affect the composition of gut microbiota, the role of gender and sex hormones has not yet been sufficiently investigated.

Mounting evidence has shown that gender and sex hormones can play a pivotal role in modulating human response to external factors, likely through a different effect on microbiota. For example, in the study by Org et al. [15], male and female mice exhibited a significant difference in the abundance of several microbial species. Interestingly, this sex-related microbiota composition explained the variability of metabolic response when mice underwent an 8-week high-fat high-sucrose diet. In addition, to determine whether these findings were mediated by sex hormones, gonadectomized and hormone-treated mice underwent the same diet. The results showed that the hormonal status affected the composition of microbiota more on the chow diet in males, whereas in females this effect was more evident after the high-fat diet. Therefore, these experiments highlighted the role of gender on targeting gut microbiota composition and the response to dietary interventions.

In other studies [16, 17], estrogens have been shown to affect gut microbiota which can, in turn, significantly influence estrogen levels. Indeed, some microbial species (also known as estrabolome) can regulate circulating estrogens through the secretion of beta-glucuronidase, a bacterial enzyme that deconjugates estrogens and phytoestrogens in their active forms which can be reabsorbed in the intestine and enter the bloodstream [18].

Dysbiosis can reduce estrabolome, and consequently, the deconjugation of estrogen and phytoestrogen into their circulating active forms with the impairment of estrogen-receptor activation [19]. This condition can induce a wide range of diseases, such as polycystic ovary syndrome (PCOS) [20], obesity and obesity-associated metabolic diseases [7, 21], cardiovascular disease (CVD) [22], cognitive decline [23], type 1 endometrial hyperplasia, and endometrial and breast cancer (BC) [24]. Moreover, estrogens regulate the microbiological environment of the female reproductive tract by maintaining epithelial thickness, glycogen levels, mucus secretion, and decreasing vaginal pH through the promotion of *Lactobacilli* colonization and lactic acid production [25]. Consequently, during menopause, the abundance of vaginal *Lactobacilli* decreases along with hormonal and epithelial changes [26]. Finally, during the normal women life cycle, menopause is characterized by a dramatic reduction in estrogens and other female sex hormones [27, 28]. Overall, this evidence suggests that the composition of the microbiota could play a pivotal role in the onset or progression of some menopause-related clinical conditions [29].

Therefore, the aim of this review was to give an overview of the relationship between microbial dysbiosis and the most common menopause-related diseases (postmenopausal osteoporosis, BC, endometrial hyperplasia, periodontitis, obesity, and CVD). In addition, evidence on the effects of probiotic supplementation in postmenopausal women was discussed to evaluate whether it might be used as a therapeutic strategy for the prevention/management of menopause-related diseases.

## Rationale for the Use of Probiotics in the Treatment of the Comorbidities Associated with Menopause

The evidence that changes in the composition of the gut microbiota might have a role in the pathogenesis of a heterogenous group of human diseases suggests that these conditions could be either prevented or ameliorated by therapeutic interventions aiming to correct gut dysbiosis [30]. The standard tool to achieve this goal is the administration of probiotics. According to the World Health Organization, probiotics are *live microorganisms that when administered in adequate amounts will confer a health benefit on the host* and this definition has been retained with only minimal grammatical changes in the consensus statement issued by the International Scientific Association for Probiotics and Prebiotics in 2016 [31]. Commercial probiotic products contain various combinations of bacteria and yeasts belonging to the following genuses: *Lactobacillus*, *Bifidobacterium*, *Saccharomyces*, *Streptococcus*, *Enterococcus*, *Escherichia*, and *Bacillus*. In many cases, these preparations also include vitamins, amino acids, or essential minerals and are marketed as dietary supplements. The basic idea behind the use of probiotics in clinics is that, upon oral administration, they could populate the gut replacing dysbiotic microorganisms and restoring the normal functional activities of gut microbiota. While this could seem an obvious consequence of probiotic therapy, the evidence that it really happens is not solid [32]. What has been observed is that, in general, the microorganisms contained in probiotics only transiently colonize the gut in a manner that is highly individually variable [33]. Long-term persistence and, even more importantly, stable changes in the resident intestinal microflora seem, instead, to occur only rarely [32]. The practical consequence of these data is that continued, long-term administration is probably required to maintain the benefits of probiotic treatment.

Different mechanisms concur to determine the beneficial effects of probiotics in different human diseases also including the comorbidities of menopause, as we will discuss in detail in the following sections. In particular, these microorganisms (1) improve gut barrier function, (2) modulate immune responses, (3) release biologically active extracellular mediators, and (4) generate biologically active substances by metabolizing either endogenous molecules or molecules taken with food (see Suez et al. [32] for a comprehensive review).

In the normal healthy gut, the intestinal epithelial cells are covered by a thin layer of mucus that they synthesize and release to form a physical and functional barrier isolating the intestinal mucosa and, more in general, the systemic circulation from the content of the gut lumen. The disruption of this barrier is an important causative factor of intestinal diseases such as inflammatory bowel disease and may grant the diffusion to distant sites of antigenic or toxic substances responsible for the genesis of non-intestinal diseases such as hepatic steatosis or parodontitis.

Probiotics may improve the intestinal barrier through different mechanisms. First, they promote the secretion and release of mucus and enhance the formation of tight junctions [34, 35]. These effects are at least partially dependent on the release of soluble mediators such as indoles, which bind to pregnane X receptors, and hydroxycis-12-octadecenoic acid, which binds to GPR40 and activates the MAPK cascade [36]. In addition, probiotics reduce dysbiotic microorganism binding to intestinal epithelial cells both by competing with them for mucosal binding sites and by reducing their number through their killing via the release of antibacterial substances such as organic acids, like acetic acid and lactic acid, and bacteriocins [37, 38]. Microbial-associated molecular patterns of probiotic microorganisms, such as flagellin, pilin surface layer protein, capsule polysaccharide, lipopolysaccharide, or lipoteichoic acid, bind to specific pattern recognition receptors, including Toll-like receptors-2, 4, and 5, not only on dendritic cells but also on epithelial intestinal cells and on M-cells, a specialized cell type involved in the transcytosis of antigens to the cells of the gut-associated lymphoid tissue [39, 40]. The binding to epithelial cells promotes the synthesis and release of defensins, and several cytokines, including interleukin (IL)-6, IL-8, IL-10, tumor necrosis factor (TNF)-α, IL-1β, and interferon (IFN)-γ, increase the formation of tight junctions and exert antiapoptotic and anti-inflammatory effects. The interaction with dendritic cell receptors regulates the differentiation of naive T cells and, ultimately, the relative balance between TH1, TH2, TH17, and Treg lymphocytes [41]. Probiotics may also regulate immune responses through the release of small soluble mediators, which are generated through the metabolism of dietary fibers. This is the case of small chain fatty acids (SCFA) [42] such as butyrate and propionate which are generated in the gut and may diffuse with general circulation to exert their immunoregulatory and anti-inflammatory effects at distant sites in particular controlling Treg expansion [43–46]. Importantly, the immunomodulating effects of probiotics are not limited to the intestinal mucosa but impact immune responses systemically as it has been demonstrated in allergic disorders [47–49]. The immunomodulating and anti-inflammatory effects of probiotics and their ability to normalize gut mucosa permeability may partly explain their beneficial effects in some of the comorbidities of menopause such as osteoporosis and parodontitis. In fact, the increase in permeability which occurs in the intestinal dysbiosis of menopause prompts the activation of Th17 lymphocytes and the release of TNF-α and RANKL, ultimately leading to enhanced osteoclastogenesis and bone resorption [50]. Similar mechanisms are effective at the level of alveolar bone where they are responsible for bone resorption and the progression of the disease [51]. By reducing the plasma levels of cytokines, probiotics may also positively impact on cardiovascular risk which is increased by systemic microinflammation [52–54]. An additional important mechanism that could be responsible for the beneficial effects of probiotics on cardiovascular risk also in menopause is related to their ability to deconjugate bile salts such as lithocholic in a reaction catalyzed by the enzyme bile salt hydrolase [55]. The resulting deconjugated bile salts cannot be recycled back to the liver as efficiently as their conjugated counterparts and this leads to higher hepatic consumption of cholesterol by liver cells to synthesize new bile salts and, ultimately, to a decrease in plasma cholesterol levels [56]. Importantly, probiotics may also improve insulin resistance via SCFA and this further contributes to reducing cardiovascular risk [57].

The decrease in cytokine release and systemic inflammation induced by probiotics by the mechanisms described above might be relevant also in explaining their beneficial effect on BC whose development and progression are promoted by the inflammatory microenvironment caused by dysbiosis [58]. Another important mechanism that could have a role in determining the proposed protective role of normal microbiota and, possibly, of probiotics on breast cancer is related to the ability of these microorganisms to produce substances with anticancer activity [59]. Normal gut microbiota synthetizes small molecules with anticancer activity such as indole derivatives, indole propionic acid, and indoxyl sulfate [60, 61]. In addition, cadaverine and the bile salt metabolite lithocholic acid may also decrease cancer cell proliferation by interacting respectively with trace amino acid receptors and TGR5/FXR [62–65]. To what extent the intake of probiotics could increase the production of these compounds with anticancer properties is still uncertain. As mentioned before, probiotics express bile salt hydrolase and increase bile salt metabolism in the gut; an increase in indole-3-propionic acid was observed in rats treated with probiotics [66] but not in children affected with type I diabetes [67]. Increased levels of polyamines have been demonstrated in the elderly after treatment with bifidobacteria-containing synbiotics [68].

As mentioned above “Reciprocal interactions between estrogens and microbiota: implications in menopause” the estrabolome controls circulating levels of estrogens by metabolizing in the gut endogenous and exogenous molecules with estrogenic activity. In particular, besides deconjugating endogenous estrogens, gut microbiota also metabolizes plant lignans, the major source of phytoestrogen in Western populations, to generate enterolignans, enterolactone, and enterodiol [69]. These metabolites have a higher bioavailability than parental compounds and are responsible for most of the systemic effect of lignans; acting as modulators of estrogen receptors, these compounds exert agonist effects in certain tissues such as the bone and antagonist effects in others such as the breast. Dysbiosis may reduce enterolignan generation, cause the loss of their agonist/antagonist effect on estrogen-dependent tissues, and consequently increase the risk of osteoporosis and BC [59, 70]. A similar bioactivating role of gut microbiota has been described also for other phytoestrogens such as ellagitannins and isoflavones, which are converted respectively into urolithins and equol; these metabolites also have potent anticancer activity independent from their effects on estrogen receptors [71–73]. It has been suggested that probiotics could restore the impaired phytoestrogen bioactivation in the dysbiotic gut and such a mechanism could partly account for their beneficial effects in menopausal comorbidities such as osteoporosis and BC and give a rationale basis for the combined treatment of these conditions with probiotics plus phytoestrogens [74]. Evidence has been reported that several probiotic microorganisms including *lactobacilli* and *bifidobacterial *may perform in vitro some of the enzymatic reactions involved in lignan and isoflavone bioactivation [75–78]. Nonetheless, the clinical studies performed so far showed inconsistent results and, therefore, the relevance of this mechanism in probiotic therapeutic effects remains uncertain [79–82].

In the next sections, we will analytically review the available evidence on the role of dysbiosis and the benefits of probiotics in the main comorbidities of menopause.

## Menopausal Dysbiosis and Osteoporosis

Osteoporosis is a clinical condition with a great impact on women's health [83]. Indeed, according to the Study of Women’s Health Across the Nation (SWAN) carried out in postmenopausal women (approximately 6 years after the last menstrual period), one in six women had one or more fractures, with a rate of 11 first fractures/1000 person/years [84].

**Fig. 1:**
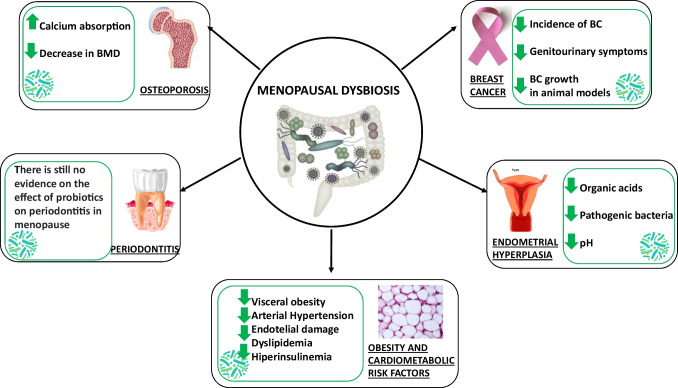
Mechanism explaining the association between dysbiosis and menopause-related diseases

Recent studies have found a strict relationship between menopause, microbiota, and bone health suggesting novel implications for the prevention and/or therapeutic strategies for osteoporosis [85, 86••] (Fig. 1).

In a double-blind, randomized, crossover acute trial carried out in 20 postmenopausal women, the addition of *Lactobacillus helveticus* to fermented milk was showed to rapidly increase serum calcium while decreasing parathormone concentrations, as compared to conventional milk [87]. These findings suggest that probiotics may promote intestinal calcium absorption. In a 12 month-double-blind, placebo-controlled study, 90 elderly women (75–80 years) with osteopenia (defined as a *t*-score between − 1 and − 2.5) were randomized to daily oral supplementation with *Lactobacillus reuteri* (LR 6475) or placebo. At the end of the study, LR 6475 reduced the loss of total volumetric body mass density (BMD) compared to placebo, thus representing a useful supplementation in elderly women with osteopenia [88]. Furthermore, in a medium-term (6 months) double-blind, randomized, clinical trial, 78 postmenopausal women at risk of osteoporosis or with untreated osteopenia were assigned to daily consumption of yoghurt enriched with bioactive compounds (calcium, vitamin D, vitamin K, vitamin C, zinc, magnesium, L-leucine) and probiotics (*Lactobacillus plantarum 3547*) or control yoghurt [89]. After 6 months, women consuming enriched yogurt showed a significantly increased BMD compared to controls. Moreover, increased N-terminal propeptide of type I collagen while decreased C-telopeptide of type I collagen concentrations—a bone formation and a bone resorption marker, respectively—were observed in the women consuming enriched yoghurt as compared to control [89].

All these studies support the feasibility and usefulness of probiotic supplementation—over the standard therapy with calcium and vitamin D—to improve bone health in menopausal women at risk of osteoporosis.

## Menopausal Dysbiosis and Breast Cancer

Scientific evidence on the association between dysbiosis and the pathogenesis of BC has been poorly investigated [90, 91•] (Fig. 1).

In a metagenomic study [92], 18 women with premenopausal BC showed no significant taxonomic differences when compared to 25 premenopausal healthy controls. However, in the same study when 44 patients with postmenopausal BC were compared to 46 healthy postmenopausal controls, 45 species differed significantly between the two groups. More in details, patients with postmenopausal BC exhibited a higher abundance of *Escherichia coli*, *Klebsiella sp_1_1_55*, *Enterococcus gallinarum*, *Actinomyces sp HPA0247*, *Shewanella putrefaciens*, and *Erwinia amylovora*, whereas there was less abundance of *Eubacterium eligens* and *Lactoisacus*. These results are in line with previous studies suggesting that the intestinal metagenomes in patients with postmenopausal BC are rich in genes that code for the biosynthesis of lipopolysaccharide that is a powerful trigger of systemic inflammation that could play a role in promoting neoplastic transformation [93]. In addition, it has been hypothesized that the microbial estrogen metabolism could play a role in the association between dysbiosis and BC. Indeed, one study demonstrated that microbiota diversity—the opposite of dysbiosis—is associated with the production of hydroxylated metabolites of estrogens in 60 postmenopausal women. In particular, compounds hydroxylated in positions 2 and 4 have been associated with a lower risk of BC [94].

As for human intervention studies, no specific studies in postmenopausal women or with probiotic supplementation on BC are available so far.

In a case–control study, 306 patients with BC and 662 healthy controls filled in a self-administered questionnaire to evaluate the consumption of beverages containing *Lactobacillus casei shirota* and of soy isoflavone-containing products (i.e., miso-soup and tofu) [95]. The survey showed that habitual consumption of *Lactobacillus casei shirota* and soy isoflavones was inversely associated with early BC incidence. Similarly, in a case–control study, 1010 patients with BC and 1950 controls were interviewed about the consumption of dairy products. The results showed that the risk of BC decreased significantly with a higher intake of yogurt, likely for the presence of probiotics [96]. Finally, a case–control study that also included a subgroup of postmenopausal women (55–64 years) reported that the consumption of fermented milk products was higher in the control group (*n* = 289) than in patients with BC (*n* = 133), suggesting a protective role in both pre- and postmenopausal women [97].

As for the potential mechanisms underlying the association between microbiota and BC, animal models demonstrated that probiotics could inhibit tumor growth and reduce tumor size, probably due to immunomodulatory, anti-angiogenesis, and anti-metastatic properties [98, 99]. Indeed, the oral administration of milk fermented by *Lactobacillus casei CRL 431* to tumor-harboring BALB/c mice produced lower rates of tumor growth, angiogenesis, and metastasis and higher survival rates among the treatment group. In addition, the cytokine profile showed decreased IL-6 and increased monocyte chemoattractant protein-1 levels, a chemotactic cytokine [43]. In a similar study, reduced concentrations of IL-10, IL-6, and mammary glands TNF-α with clinical improvements (i.e., reduced tumor growth and angiogenesis) were detected after probiotic supplementation [99].

Of note, probiotic supplementation was effective also on the improvement of genitourinary symptoms in women treated for BC as consequences of chemotherapy and estrogen deprivation [100].

A 2-week supplementation with four *Lactobacillus species* (2 capsules/day) positively influenced the colonization of vaginal microbiota (evaluated by Nugent score) in 22 postmenopausal patients with BC receiving chemotherapy [101].

Therefore, probiotic supplementation seems to have a potential role both in the prevention of BC and in the management of chemotherapy-induced side effects in BC. However, more clinical studies are needed to elucidate their efficacy and safety.

## Menopausal Dysbiosis and Type 1 Endometrial Hyperplasia

Type 1 endometrial hyperplasia is a precancerous condition characterized by a non-physiological and non-invasive endometrial growth, sustained by an increased estrogen/progesterone ratio [102]. During the fertile age, the risk of endometrial hyperplasia is associated with intermittent or absent ovulation, as PCOS. After menopause, endometrial hyperplasia is more common in women with estrogen-increasing conditions, such as obesity or hormone replacement therapy (HRT).

Endometrial hyperplasia could be also influenced by vaginal microbiota [103] (Fig. 1). Menopause is known to increase vaginal pH—due to the lack of estrogen, thus targeting microbial colonization. Interestingly, in endometrial carcinoma induced by type 1 endometrial hyperplasia, the uterine microbiota is characterized by the presence of *Atopobium vaginae* and *Porphyromonas sp*. with a pH > 4.5 [104]. These findings raise the possibility of (1) further investigating the microbiome role in the etiology or progression of endometrial cancer and (2) targeting specific bacterial strains that could favor lowering vaginal pH to reduce potentially pathogenic bacteria in the urogenital tract.

Some in vitro studies have shown that *Lactobacillus rhamnosus* BPL005 reduces pH levels by producing lactic acid and other organic acids, thus preventing endometrial infections by inhibiting some microbial species (i.e., *Atopobium vaginae*, *Gardnerella vaginalis*, *Propionibacterium acnes*, and *Streptococcus agalactiae*) [105].

However, meager evidence is available from human studies.

In a recent study, 130 healthy postmenopausal women suffering from menopausal symptoms were randomized to receive (a) 60 mg of soy isoflavones and 1 billion spores of *Lactobacillus sporogenes* or (b) calcium and vitamin D3 for 1 year. At the end of the study, menopausal symptoms significantly improved in the group consuming soy isoflavones plus *Lactobacillus sporogenes* versus the other group (calcium plus vitamin D3). No differences in endometrial thickness between groups were observed [106]. This study suggested that lactic bacteria might improve the absorption of soy isoflavones through the hydrolyzation of genistin and daidzin into the active aglycons by glycosidases, thus increasing the bioavailability of soy isoflavones.

## Menopausal Dysbiosis and Periodontitis

Postmenopausal women have shown an increased risk of xerostomia (dry mouth), tooth mobility, and periodontitis (infection of the gums), likely related to reduced estrogen levels [29]. Indeed, oral mucosa and salivary glands present estrogen receptors and hypoestrogenemia has shown to activate polymorphonucleated and lymphocytes, increase cytokine levels, and modify the oral microbiota with an increase in gram-negative bacteria. In particular, some bacterial species such as *Porphyromonas gingivalis* and *Tannerella forsythensis* have been specifically associated with periodontitis in postmenopausal women [107]. Moreover, a 2-year open follow-up study in 400 postmenopausal women aged 50–58 years investigated the association between HRT and the composition of oral microbiota. After 2 years, in postmenopausal women on HRT (*n* = 200), there was a significant reduction in the abundance of *Porphyromonas gingivalis* and *Tannerella forsythensis* compared to the baseline. Conversely, in contrast, no changes in the oral microbiota were observed in the control group (*n* = 200) not treated with HRT [108]. Although some bacterial species have been specifically associated with periodontitis in postmenopausal women, to date, there is no evidence on the effect of probiotics in the prevention and treatment of periodontitis in this target group (Fig. 1). Therefore, further studies are required to evaluate whether probiotic supplementation could represent a useful strategy to modulate the oral microbiota in postmenopausal women.

## Menopausal Dysbiosis and Obesity

Menopause is highly associated with obesity, and increased adiposity is the main risk factor for increased cardiometabolic alterations in postmenopausal women [109, 110]. Indeed, a 4-year observational study investigated changes in body weight and body fat during the menopausal transition in 156 healthy perimenopausal women. The results showed that subcutaneous abdominal fat increased in all participants; however, only women who enter menopause had a significant increase in visceral abdominal fat suggesting a redistribution of body fat mostly as central adiposity [111].

A recent meta-analysis of 11 longitudinal studies (*n* = 2.472 women) where participants were premenopausal at baseline and postmenopausal at follow-up highlighted significant differences in body weight and body fat distribution between premenopausal and postmenopausal periods. More in detail, as compared to baseline (premenopausal period), body mass index (BMI), percentage of body fat, waist and hip circumference, and visceral and trunk fat significantly increase in postmenopausal women [112]. In a more recent prospective cohort study with a 15-year follow-up, menopause and aging were independently correlated with increased BMI in 929 women who entered menopause during follow-up [113].

Body fat accumulation during menopause seems to be related to several mechanisms, including hormonal imbalance, reduction of energy expenditure, sedentary life, and increase in food intake [28].

Animal experiments demonstrated that the murine model of menopause (ovariectomized rats) had increased body weight and visceral fat [114], potentially due to increased food intake [115], decreased lipolysis [116], and reduced energy expenditure [117]. In particular, the expression of uncoupling proteins (UCPs) in brown and white adipose tissue could play a role in estrogen-mediated changes in body weight and energy expenditure. As a matter of fact, ovariectomized rats have a decreased UCP1 and UCP2 expression in brown and white adipose tissue, respectively, which translates into reduced energy expenditure [117]. Interestingly, when ovariectomized Sprague–Dawley rats were treated with estrogen, they exhibited a reduced weight gain and intra-abdominal fat accumulation. Nevertheless, estrogen therapy has been shown to induce uterine hypertrophy in the mouse model that makes it unsuitable for the prevention of weight gain in postmenopausal women [118].

As for the relationship between obesity and microbiota composition, the *Firmicutes/Bacteroidetes ratio* was directly associated with BMI in both animal models and studies in humans [119, 120]. In particular, women with obesity had a higher *Firmicutes/Bacteroidetes ratio* than men and increased plasma concentration of bacterial lipopolysaccharide—a well-known mediator of systemic inflammation [121]. Furthermore, it has been shown that obesity might affect the metabolic activity of some microbial species, including the hydrolyzation of isoflavones, that exhibit estrogen-like properties. As an example, a cross-sectional study of 355 women with overweight and obesity (*n* = 137 peri- and *n* = 218 postmenopausal women) who consumed at least 3 servings/week of soy (a source of isoflavones) demonstrated that women with higher BMI exhibited lower urinary concentrations of daidzein and its metabolites (equol and O-desmethylangolensin). This finding suggests an association between obesity and the alteration of microbiota composition and activity [122].

Overall, evidence available so far on the link between menopause, obesity, and microbiota composition is rather scarce. However, it rises some intriguing insights on novel strategies for body weight control tailoring microbial species that can metabolize estrogens and compound with estrogen-like properties.

## Menopausal Dysbiosis and Cardiometabolic Risk

As mentioned in the previous section, menopause-related central obesity is a risk factor for cardiometabolic diseases [28, 123]. Indeed, menopause and early hormonal deprivation have been independently associated with a higher risk of metabolic syndrome, CVDs, stroke, heart failure, and total and heart disease mortality [123].

Over obesity, postmenopausal women exhibit a microbiota-dependent production of metabolites that may increase their cardiometabolic risk. More in detail, a metagenomic study in postmenopausal women demonstrated a strict association between several gut microbial species (i.e., *Clostridium bolteae*, *Eubacterium ramulus*, *Ruminococcus torques*, *Catenibacterium mitsuokai*, *Holdemanella biformis*) and markers of insulin resistance, dyslipidemia, and inflammation, independently from body weight [124].

Human intervention studies with probiotic supplementation in postmenopausal women have already shown a favorable effect on some cardiovascular risk factors. In a 12-week randomized placebo-controlled trial, 81 Caucasian women with obesity were assigned to a low dose or a high dose of a probiotic containing *Bifidobacterium* and *Lactobacillus*. At the end of the study, high dose significantly improved endothelial dysfunction, systolic blood pressure, and markers of inflammation (IL-6, TNF-α) and angiogenesis (vascular endothelial growth factor and thrombomodulin) [125]. In a similar study, high dose significantly improved body fat (waist circumference, fat mass, subcutaneous fat) and metabolic markers (uric acid, total cholesterol, triglycerides, low-density lipoprotein cholesterol, glucose, insulin, and homeostatic model assessment for insulin resistance) [126].

Several mechanisms could explain the pleiotropic effects of probiotic supplementation on multiple cardiometabolic risk factors [127, 128] (Fig. 1). Indeed, it is known that microbiota has a pivotal role in maintaining the integrity of the intestinal barrier, thus reducing bacteria translocation and, consequently, systemic inflammation. On the other hand, from the fermentation of polysaccharides and undigested proteins, some microbial species can produce SCFA (namely acetate, propionate, and butyrate), which can influence several metabolic pathways. Briefly, SCFA act as a mediator of transcriptional regulations and post-translational modifications, by the inhibition of lysine and histone deacetylase, thus influencing important transcription factors (in particular, peroxisome proliferator-activated receptor γ and aryl hydrocarbon receptor). SCFA can also activate signaling transduction pathways, thus activating multiple free fatty acid receptors.

Although further studies are needed, supplementation with probiotics could represent a useful and safe tool to control several cardiometabolic risk factors in postmenopausal women.

## Probiotic and Prebiotic Safety and Study Limitations

Human supplementation with probiotics and prebiotics is usually considered to be safe [129]. However, although probiotics and prebiotics are generally considered safe in healthy adults, their use has been linked to a higher risk of infection and/or morbidity in critically ill adults in intensive care units, and postoperative, hospitalized, or immunocompromised patients [129]. So far, few cases of bacteremia, sepsis, and endocarditis caused by *L*. *rhamnosus* GG or *L*. *casei* lactobacilli have been reported [130]. Infections with *Bifidobacteria* are considered rare. However, bacteraemias, sepsis, and cholangitis induced by *Bacillus subtilis* [131] and fungal sepsis caused by *Saccharomyces boulardii* [132] have been reported. Of note, the association between probiotic and prebiotic use and increased risk of infection in immunocompromised patients needs to be further evaluated [133]. Overall, probiotic and prebiotic supplementation is considered safe in general when administered to immunocompetent individuals.

Certainly, the currently available studies on probiotics and prebiotics have a number of limitations that cannot be underestimated when drawing conclusions on their use. For instance, many studies have evaluated populations that are too small or have considered durations of use that are too short. Another limitation is the lack of microbial analyses of feces, which could demonstrate the influence of probiotic bacteria on the composition of the gut microbiota. It would also be interesting to perform mechanistic studies (similar to animal models) to explain the favorable effects of the metabolic activity of probiotics and prebiotics.

## Conclusions

Table 1 summarizes the randomized studies reported on the use of probiotics in postmenopausal women. Although evidence from human intervention studies is limited so far, probiotic supplementation in postmenopausal women could represent a feasible and safe strategy to manage the menopause-related disease. In particular, oral probiotic formulations—especially those including *Lactobacillus* ssp. *casei*, *helveticus*, *rhamnosus*, and *reuteri*—might have pleiotropic beneficial effects on health by:

- Promoting intestinal calcium absorption and reducing a further decrease in BMD in women at risk of osteoporosis or with osteopenia, thus potentially delaying bone damage
- Reducing the incidence of BC and by improving the genitourinary symptoms associated with BC therapy
- Promoting the reduction of vaginal pH, through the production of organic acids and the reduction of pathogenic bacteria which are risk factors for type 1 endometrial hyperplasia in in vitro models
- Improving insulin resistance, dyslipidemia and inflammation, thus reducing the cardiometabolic risk of the postmenopausal woman

**Table 1:** Randomized controlled trials of probiotics in postmenopausal women

**References**	**Design**	**Subjects**	**Probiotics strains (amount)**	**Probiotic form and dose**	**Duration**	**Outcomes**	**Main findings**
Lambert et al. [74]	Randomized, double-blind, placebo-controlled trial	78 postmenopausal women with osteopenia (40 probiotic; 38 placebo)	Heterogeneous culture (proprietary) of probiotic lactic acid bacteria	Powder twice daily	12 months	Primary: effect of red clover extracts rich in isoflavone aglycones and probiotics against bone mass density loss. Secondary: effects of red clover extract on bone turnover markers, estrogen metabolites, plasma isoflavone concentration, equol-producer status, plasma lipid concentrations, and blood pressure	Red clover extracts significantly attenuated bone mineral density loss at the L2–L4 lumbar spine vertebra, femoral neck, and trochanter compared with the control group. Plasma concentrations of collagen type 1 cross-linked C-telopeptide were significantly decreased in the red clover extract group compared with the control group. Red clover extracts significantly elevated plasma isoflavone concentration, urinary 2-hydroxyestrone (2-OH) to 16a-ydroxyestrone (16a-OH) ratio, and equol-producer status compared with the control group
Nettleton et al. [82]	Randomized, crossover trial	40 postmenopausal women (20 with no cancer history; 20 breast cancer survivors)	*Lactobacillus acidophilus* DDS1 and *Bifidobacterium longum* (10^9^ CFU)	Capsules once daily	6 weeks	Effect of probiotic consumption together with soy protein on the bioavailability and plasma concentrations of phytoestrogens, particularly the isoflavone metabolite equol	Plasma phytoestrogen concentrations and the number of equol-producers did not differ between the soy and soy plus probiotics diets
Narva et al. [87]	Randomized, double-blind, crossover trial	20 postmenopausal women	*Lactobacillus helveticus* (not reported)	Fermented milk (220 ml) or peptide orange juice (400 ml) once daily	22 days	Effect of milk fermented with *L. helveticus* and small peptides formed by the bacterium on acute changes in calcium metabolism and bone resorption in postmenopausal women	*L. helveticus*-fermented milk reduced serum parathormone and increased serum calcium compared to the control milk. *L. helveticus*-derived peptides had no significant acute effect on calcium metabolism; in fact, ionized calcium was lower and parathormone higher after the juice containing peptides compared to the control juice
Nilsson et al.[88]	Randomized, double-blind, placebo-controlled trial	70 postmenopausal women with low bone mass density (34 probiotic; 36 placebo)	*Lactobacillus reuteri* ATCCPTA 6475 (5 × 10^9^ CFU)	Stick packs (freeze-dried probiotics mixed with maltodextrin powder) twice daily	12 months	Primary: changes after 12 months in tibia total volumetric bone mass density. Secondary: relative changes after 12 months in areal bone mass density measured at the hip and spine; trabecular bone volume fraction; cortical volumetric bone mass density; cortical thickness; serum markers for bone turnover; serum markers for inflammation; serum glycated hemoglobin; and body composition	*L. reuteri* 6475 reduced loss of total volumetric bone mass density compared to placebo both in the intention-to-treat analysis and per protocol analysis
Morato-Martínez et al. [89]	Randomized, double-blind, placebo-controlled trial	65 postmenopausal women at risk of osteoporosis or untreated osteopenia (33 probiotic; 32 placebo)	*Lactobacillus plantarum* 3547 (10^10^ CFU)	Mix packs with water (120 ml) and lyophilized product (30 g) separated by a membrane once daily	24 weeks	Effect of regular consumption of a dairy product enriched with bioactive nutrients (calcium, vitamin D, vitamin K, vitamin C, zinc, magnesium, L-leucine, and *L. plantarum* 3547) on bone metabolism markers of postmenopausal women at risk of osteoporosis	The intervention group showed a significantly increased bone mass compared to the control group. The intervention group maintained bone mass density compared to the control group, whose bone mass density significantly decreased at the end of the study. For biochemical markers, the intervention group significantly increased serum levels of the N-terminal propeptide of type I collagen bone formation marker and decreased the carbo-terminal telopeptide of type I collagen bone resorption marker compared to the control group
Marschalek et al.[101]	Randomized, double-blind, placebo-controlled pilot trial	22 postmenopausal women with breast cancer undergoing chemotherapy (11 probiotic; 11 placebo)	*Lactobacillus crispatus* LbV 88, *Lactobacillus rhamnosus* LbV 96, *Lactobacillus jensenii* LbV 116, *Lactobacillus gasseri* LbV 150N (2.5 × 10^9^ CFU for each one)	Capsules twice daily	2 weeks	Effect of an orally administered preparation of 4 *L*. spp. on the vaginal microbiota of postmenopausal women with breast cancer undergoing chemotherapy	There was a positive influence on the vaginal microbiota in 7/11 (63%) women in the intervention group and 4/11 (36%) women in the control group. There was a shift in the Nugent score towards normal microbiota levels in the intervention group and a significant deterioration of the Nugent score in the control group
Colacurci et al. [106]	Randomized, placebo-controlled trial	124 healthy postmenopausal women suffering from menopausal symptoms (62 probiotic; 62 placebo)	*Lactobacillus sporogenes* (1 bilion spores)	Tablets once daily	12 months	Effect of a nutraceutical compound containing isoflavones and *L. sporogenes* compared to calcium and vitamin D alone on endometrium, breast, and liver function	After 12 months of treatment mammographic density, endometrial thickness and hepatic function did not show significant differences between groups, while menopausal symptoms were progressively and significantly reduced in severity and frequency during treatment with soy isoflavones plus *L. sporogenes* vs calcium plus vitamin D3
Szulińska et al.[125]	Randomized, placebo-controlled trial	81 postmenopausal women with obesity (23 high probiotic dose; 24 low probiotic dose; 24 placebo)	*Bifidobacterium bifidum* W23, *Bifidobacterium lactis* W51, *Bifidobacterium lactis* W52, *Lactobacillus acidophilus* W37, *Lactobacillus brevis* W63, *Lactobacillus casei* W56, *Lactobacillus salivarius* W24, *Lactococcus lactis* W19, and *Lactococcus lactis* W58 (2.5 × 10^9^ CFU for low-dose group; 10^10^ CFU for high-dose group)	Powder twice daily	12 weeks	Primary: effects of different doses of supplemented multispecies probiotics on the functional parameters of endothelial and vascular dysfunction. Secondary: effects of different doses of supplemented multispecies probiotics on the biochemical parameters of endothelial and vascular dysfunction	High doses of probiotic supplementation decreased systolic blood pressure, vascular endothelial growth factor, pulse wave analysis systolic pressure, pulse wave analysis pulse pressure, pulse wave analysis augmentation index, pulse wave velocity, interleukin-6, tumor necrosis factor alpha, and thrombomodulin. Low doses of probiotic supplementation decreased systolic blood pressure and interleukin-6 levels. The mean changes in the estimated parameters, compared among the three groups, revealed significant differences in vascular endothelial growth factor, pulse wave analysis systolic pressure, pulse wave analysis augmentation index, pulse wave velocity, tumor necrosis factor alpha, and thrombomodulin
Szulińska et al. [126]	Randomized, placebo-controlled trial	81 postmenopausal women with obesity (23 high probiotic dose; 24 low probiotic dose; 24 placebo)	*Bifidobacterium bifidum* W23, *Bifidobacterium lactis* W51, *Bifidobacterium lactis* W52, *Lactobacillus acidophilus* W37, *Lactobacillus brevis* W63, *Lactobacillus casei* W56, *Lactobacillus salivarius* W24, *Lactococcus lactis* W19, and *Lactococcus lactis* W58 (2.5 × 10^9^ CFU for low-dose group; 10^10^ CFU for high-dose group)	Powder twice daily	12 weeks	Primary: effects of different doses of supplemented multispecies probiotics on the lipopolysaccharide level. Secondary: effects of different doses of supplemented multispecies probiotics on cardiometabolic parameters	There were significant favorable changes (mostly large or medium effects) in evaluated parameters in both the high- and low-dose groups but not in the placebo group. In the high-dose group, lipopolysaccharide, waist, fat mass, subcutaneous fat, uric acid, total cholesterol, triglycerides, low-density lipoprotein cholesterol, glucose, insulin, and insulin-resistant index were improved. Similar changes were observed in the low-dose group, except for lipopolysaccharide, uric acid, triglycerides, and glucose levels. Additionally, significant differences were observed in both groups in terms of fat percentage and visceral fat. When the mean changes were compared between the three groups, statistically significant differences in lipopolysaccharide levels, uric acid, glucose, insulin, and insulin-resistant index were found

*CFU* colony-forming unit

## Abbreviations

PCOS: Polycystic ovary syndrome
CVD: Cardiovascular disease
BC: Breast cancer
IL: Interleukin
TNF: Tumor necrosis factor
IFN: Interferon
SCFA: Short-chain fatty acids
BMD: Body mass density
HRT: Hormone replacement therapy
BMI: Body mass index
UCP: Uncoupling protein
